# Preclinical Testing of an Oncolytic Parvovirus: Standard Protoparvovirus H-1PV Efficiently Induces Osteosarcoma Cell Lysis In Vitro

**DOI:** 10.3390/v9100301

**Published:** 2017-10-17

**Authors:** Carsten Geiss, Zoltán Kis, Barbara Leuchs, Monika Frank-Stöhr, Jörg R. Schlehofer, Jean Rommelaere, Christiane Dinsart, Jeannine Lacroix

**Affiliations:** 1 Division of Tumor Virology, Program Infection, Inflammation and Cancer, German Cancer Research Center (DKFZ), Im Neuenheimer Feld 242, 69120 Heidelberg, Germany; cargeiss@uni-mainz.de (C.G.); z.kis10@imperial.ac.uk (Z.K.); b.leuchs@dkfz-heidelberg.de (B.L.); joteres@arcor.de (J.R.S.); j.rommelaere@dkfz-heidelberg.de (J.R.); c.dinsart@dkfz-heidelberg.de (C.D.); 2 Department of Bioengineering, Imperial College, London W7 2AZ, UK; 3 Institute for Integrated Economic Research, London W5 2NT, UK; 4 Division of Viral Transformation Mechanisms, Program Infection, Inflammation and Cancer, German Cancer Research Center (DKFZ), Im Neuenheimer Feld 242, 69120 Heidelberg, Germany; frank-stoehr.monika@t-online.de; 5 Department of Pediatric Hematology, Oncology and Immunology, Center for Pediatric and Adolescent Medicine, University Hospital, Im Neuenheimer Feld 430, 69120 Heidelberg, Germany

**Keywords:** osteosarcoma, protoparvovirus H-1 (H-1PV), oncolytic virus, viral cytotoxicity

## Abstract

Osteosarcoma is the most frequent malignant disease of the bone. On the basis of early clinical experience in the 1960s with H-1 protoparvovirus (H-1PV) in osteosarcoma patients, this effective oncolytic virus was selected for systematic preclinical testing on various osteosarcoma cell cultures. A panel of five human osteosarcoma cell lines (CAL 72, H-OS, MG-63, SaOS-2, U-2OS) was tested. Virus oncoselectivity was confirmed by infecting non-malignant human neonatal fibroblasts and osteoblasts used as culture models of non-transformed mesenchymal cells. H-1PV was found to enter osteosarcoma cells and to induce viral DNA replication, transcription of viral genes, and translation to viral proteins. After H-1PV infection, release of infectious viral particles from osteosarcoma cells into the supernatant indicated successful viral assembly and egress. Crystal violet staining revealed progressive cytomorphological changes in all osteosarcoma cell lines. Infection of osteosarcoma cell lines with the standard H-1PV caused an arrest of the cell cycle in the G2 phase, and these lines had a limited capacity for standard H-1PV virus replication. The cytotoxicity of wild-type H-1PV virus towards osteosarcoma cells was compared in vitro with that of two variants, Del H-1PV and DM H-1PV, previously described as fitness variants displaying higher infectivity and spreading in human transformed cell lines of different origins. Surprisingly, wild-type H-1PV displayed the strongest cytostatic and cytotoxic effects in this analysis and thus seems the most promising for the next preclinical validation steps in vivo.

## 1. Introduction

In children and adolescents, cancers are the diseases with the highest mortality [1,2]. Osteosarcoma is the most common type of primary bone cancer and the eighth most common form of pediatric cancer [3]. Current treatment strategies, introduced in the 1980s, include neo-adjuvant chemotherapy, resection of all primary and metastatic lesions, and subsequent adjuvant chemotherapy [4,5,6]. These strategies achieve average 5-year survival rates of 60–70%. Approximately 20% of osteosarcoma patients initially present with metastatic disease, predominantly in the lungs. In metastatic osteosarcoma patients, the 5-year survival rate is drastically reduced to 15–30% [6,7]. Especially for patients with high-risk or metastatic disease, novel treatment approaches are urgently needed. Among the targeted therapies now available to refractory or relapsed osteosarcoma patients and having shown first survival benefits [8], immunomodulatory approaches stimulating the antitumor immune response are considered the most promising [9,10].

Oncolytic viruses are a class of promising, potent, and highly specific anticancer agents, combining specific cytotoxicity towards transformed cells with an immunotherapeutic action [11,12,13]. These viruses induce lytic infection of malignant cells and thereby stimulate the innate and adaptive immune systems by promoting the availability of tumor antigens which initiate cross-priming and vaccination effects [11,14]. The use of such viruses can also be synergistically combined with other treatment modalities inducing immunogenic cancer cell death [15]. The efficacy of several oncolytic viruses has been proven through in vivo preclinical osteosarcoma studies. In a study where wild-type oncolytic Seneca Valley Virus was tested on six human osteosarcoma–xenograft severe combined immuno-deficient mouse models, only two models showed significantly extended event-free survival and no model displayed any effect on overall survival [16]. In another study, the wild-type Semliki Forest Virus was found to increase dramatically the survival of osteosarcoma–xenograft nude mice [17]. In xenograft mice with localized or metastatic osteosarcoma, the recombinant, conditionally replicating adenoviruses Ad5-Δ24RGD, Ad-OC-E1a, and Adenovirus VCN-01 significantly delayed tumor growth (localized tumors) or reduced number of metastatic lesions (pulmonary metastases) [18,19,20,21]. In the first phase I clinical trial of the naturally occurring reovirus isolate Reolysin in pediatric sarcoma patients (including osteosarcoma patients), safety data have been published [22].

The rodent protoparvovirus H-1 (H-1PV) is another promising therapeutic agent. Its clinical safety upon intratumoral or intravenous injection has been demonstrated in a recently published first phase I/IIa clinical trial recruiting adult glioblastoma patients [23]. H-1PV is a wild-type oncolytic virus occurring naturally in rats. A natural tropism of this virus for the developing skeleton has been hypothesized, as infection in utero in embryonic rodents induces craniofacial dysmorphisms in successfully infected animals [24]. First-in-man applications of this wild-type rodent parvovirus in two adolescent osteosarcoma patients in the 1960s were prompted by the discovery that has oncolytic potential [25,26]. In this compassionate use study, no severe virus-induced toxicity was reported. Thereafter, more than four decades elapsed before the first phase I virotherapy trials with other oncolytic viruses started to recruit osteosarcoma patients [27]. To prepare for a clinical trial of H-1PV in osteosarcoma patients, systematic preclinical research is needed.

Protoparovirus H-1PV consists of an approximately 5.1-kb single-stranded DNA genome enclosed in a 25-nm non-enveloped icosahedral shell. The viral genome contains two main transcriptional units: one encoding the non-structural (NS) proteins and one encoding the viral capsid proteins (VP) and the small alternatively translated (SAT) [27,28]. The major large non-structural protein NS1 is essential to virus replication and cytotoxicity in permissive cells [28].

In H-1PV-infected animals and humans, the immune system produces virus-specific antibodies 5–7 days after infection, and these at least partially neutralize the virus [25,26]. Additionally, H-1PV triggers anticancer vaccination effects, whereby animals cured of cancer by the oncolytic virus remain immune to the same malignant disease even in the absence of the virus [29].

In recent years, H-1PV fitness mutants have been isolated and tested for antineoplastic efficacy [30]. It has been hypothesized that the greater egress of infective viral progeny viruses and the higher infectivity observed in vitro with these mutants may lead to higher antineoplastic efficacy in vivo. This has been confirmed for Del H-1PV in pancreatic cancer and cervix carcinoma xenograft models [30]. 

Here, as a first step in assessing the responsiveness of osteosarcomas to parvovirus treatment, preclinical testing of wild-type H-1PV and of two derived mutants, Del H-1PV, and DM H-1PV, has been performed in vitro.

## 2. Materials and Methods

### 2.1. Ethics Statement

Human neonatal foreskin fibroblasts were supplied by CET celleng-tech (Coralville, IA, USA). Primary human osteoblasts were obtained from PromoCell GmbH (Heidelberg, Germany). All other osteosarcoma cell lines (CAL 72, H-OS, MG-63, SaOS-2, and U2-OS) were obtained from Cell Line Service GmbH (Eppelheim, Germany). The WAC-2 clone, derived from the neuroblastoma cell line SH-EP by stable transfection, contains an ectopic Cytomegalovirus N-myc proto-oncogene *(CMV-MYCN)* enhanced expression cassette [31] was kindly provided by Prof. Dr. med. O. Witt, Clinical Cooperation Unit Pediatric Oncology, German Cancer Research Center (Heidelberg, Germany). For data confirmation, a second batch was obtained from Prof. A. Schramm, Department of Pediatric Hematology and Oncology, University Hospital Essen (Essen, Germany). 

### 2.2. Mammalian Cell Culture

All cell cultures were maintained in 5% CO_2_ at 37°C and 100% relative humidity. Human neonatal foreskin fibroblast cells were propagated in Human Foreskin Fibroblast Expansion Medium (Cellular Engineering Technologies, Coralville, IA, USA) containing 10% fetal calf serum (FCS), 100 U/mL penicillin, and 100 μg/mL streptomycin. Non-transformed human osteoblasts were grown in Osteoblast Growth Medium (PromoCell GmbH, Heidelberg, Germany). The culture medium for osteosarcoma cell lines was Dulbecco’s Modified Eagle’s Medium (DMEM) or Minimum Essential Medium (MEM) for H-OS cells, supplemented with 2 mM L-glutamine, 10% fetal bovine serum, 100 U/mL penicillin, and 100 μg/mL streptomycin (final concentrations). The human neuroblastoma cell line WAC-2 was cultured in Roswell Park Memorial Institute (RPMI-1640) medium containing 10% fetal bovine serum, 100 U/mL penicillin, and 100 μg/mL streptomycin. For passaging, cells were detached in 0.05% or 0.25% Trypsin-EDTA solution and then resuspended in fresh culture medium. All cell lines and non-transformed cell cultures were routinely checked for contamination [32] and genomic identity [33], using previously established methods. Osteosarcoma cell lines used in this study are listed in Table S1.

### 2.3. Viruses and Virus Production

Wild-type H-1 parvovirus (H-1PV) and the recombinant H-1 parvovirus (Chi-hH-1/EGFP) expressing enhanced green fluorescent protein (EGFP) were produced at the Virus Production & Development Unit, Division of Tumor Virology, German Cancer Research Center, Germany. The recombinant parvovirus Chi-hH-1/EGFP was obtained by co-transfecting HEK-293T cells with the corresponding recombinant vector DNA and a helper plasmid expressing the viral capsid genes in trans [34]. It was purified in the same manner as the wild-type H-1PV. H-1PV was produced by infecting human newborn embryonic kidney NBK-324K cells at a multiplicity of infection (MOI) of 10^−2^ plaque-forming units per cell (PFU/cell). Four to five days after infection, the crude virus was extracted from infected cells and purified by filtration (pore diameter: 0.2 μm) and by iodixanol gradient centrifugation as previously described [35]. Contamination of virus stocks with endotoxins was below 2.5 U/mL. The Del H-1PV mutant was produced as previously described [30].

### 2.4. Detection of Infectious H-1PV Particles

Viral titers were determined by means of infected cell hybridization assays or by plaque assay as previously described [36]. Titration experiments were carried out in triplicates. For the hybridization assay, NB-324K cells (7.6 × 10^3^ cells/well) were seeded into 96-well plates. The cells, 24 h after seeding, were infected with 10-fold serial dilutions of the virus sample and incubated for 72 h under 5% CO_2_, at 37 °C and 100% relative humidity. Next, the cells were lysed with 0.75 M NaOH. The DNA was transferred to a nylon membrane, UV-cross-linked, hybridized with a ^32^P-labeled NS-specific DNA fragment, and used to expose X-ray films for autoradiography.

### 2.5. Western Blot Analysis

Western blotting was performed as described [37]. The cells, 12 h after seeding, were either mock-infected or exposed to wild-type H-1PV (MOI: 1 PFU/cell). Osteosarcoma cells were harvested at 24-h intervals over a 5-day period post-infection. Briefly, approximately 10^6^ mock- or H-1PV-infected cells were harvested at the time points indicated, collected by centrifugation, and washed with phosphate-buffered saline (PBS). Cell pellets were kept on ice for 1 h in RIPA lysis buffer with freshly added protease inhibitor (complete Mini, EDTA-free, Roche Diagnostics, Indianapolis, IN, USA) and then centrifuged at 15,000× *g* for 10 min at 4 °C. The supernatants were stored at −80 °C until further analysis. Protein concentrations of the cell lysates were determined photometrically with the Pierce^TM^ BCA Protein Assay Kit (Thermo Fisher Scientific, Waltham, MA, USA), according to the manufacturer’s protocol. Twenty micrograms of each cell lysate was resolved by 10% sodium dodecyl sulfate polyacrylamide gel electrophoresis (SDS-PAGE) and transferred to a nitrocellulose membrane (Schleicher & Schüll, Kassel, Germany).

The following antibodies were used: monoclonal mouse anti-actin clone C4 (MP Biomedicals, Illkirch, France), rabbit polyclonal antiserum MK3 raised against the viral NS1 protein, kindly provided by N. Salomé, INSERM U1109, Strasbourg, France [36]. Binding of antibodies, detection with horseradish peroxidase-conjugated anti-rabbit or anti-mouse IgGs (dilution 1:5000), and chemiluminescence assays were performed as previously described [38].

### 2.6. Microscopy

Comparative fluorescence and phase contrast images were recorded under an Olympus CKX41 inverted phase contrast microscope (Olympus Corporation, Tokyo, Japan) using the Cell B software from Olympus. Phase contrast images were captured with the Keyence BZ 9000 microscope (KEYENCE Microscope Europe NV/SA, Mechelen, Belgium) and the imaging softwares BZ II Viewer and BZ II Analyzer supplied with it by the manufacturer.

### 2.7. Viral DNA Extraction and Quantitative Real-Time Polymerase Chain Reaction (qPCR)

One-tenth of the total volume, i.e., 1 mL, of the culture medium of adherently growing cells was collected from each cell culture dish at regular intervals post-infection before harvesting the cells for protein extraction and subsequent western blot analysis (see Section 2.5). This supernatant was subjected to alkaline lysis in 1 M NaOH in TE buffer for 30 min at 56 °C.

After neutralization, samples were diluted 1:100 in sterile water. Quantification of viral DNA in these solutions was carried out by real-time qPCR with an NS1-specific TaqMan^TM^ probe (from Applied Biosystems, Thermo Fisher Scientific), according to previously published procedures [31].

### 2.8. Cell Viability and Cell Death Assessment

Proliferation of bone tumor cells was tested in a 96-well plate format as published, using 3-(4,5-dimethylthiazol-2-yl)-2,5-diphenyltetrazolium bromide (MTT) as a test reagent (from Sigma-Aldrich^®^, Inc., St. Louis, MO, USA) [39]. To assess the viability of osteosarcoma cell lines, cells were seeded at 2,500 cells per well and the assays were performed on days 3 and 6 post-infection. For the MTT tests pertaining to the positive control cell line (WAC-2 neuroblastoma cells), 1000 cells per well were seeded and MTT-tests were performed on days 3 and 6 post-infection. To determine the viability of primary osteoblasts and fibroblasts on day 7 after infection, cells were seeded into 96-well plates at 2500 cells per well. 12 h after cell seeding, the medium was removed and virus inoculum or buffer was added to the cells in 50 μL serum-free medium at the indicated MOI. Two hours after infection, 50 μL culture medium supplemented with 20% FCS was added in order to achieve culture conditions appropriate for the periods mentioned above. At the end of the infection period, cells were incubated for 1 h with 0.5 μg/mL MTT solution. After removing the solution, the cells were allowed to dry and isopropanol was added at 100 μL per well. Absorbance values were photometrically determined at 570 nm with a Multiscan Plus^TM^ Microplate Reader (from Titertek Instruments, Huntsville, AL, USA).

Cell lysis was determined by the amount of lactate dehydrogenase (LDH) released into the culture medium. For this, the Cytotox 96 cytotoxicity assay kit^TM^ (from Promega, Mannheim, Germany) was used according to the manufacturer’s instructions. The absorbance at 490 nm of red formazan generated by an LDH-catalyzed reaction was measured with the Multiscan Plus^TM^ Microplate Reader. Both cell viability tests and cell lysis assays were carried out in five replicates. The median lethal dose (LD50) of input virus was defined as the MOI at which cell viablitiy was reduced by at least 50% (as determined by the MTT test) and cell lysis reached at least 50% (as determined in the LDH release assay).

### 2.9. Flow Cytometric Characterization of the Cell-Cycle Distribution of Cells and of the Sub-G1 Apoptotic Cell Population

Osteosarcoma cells or WAC-2 neuroblastoma cells were seeded in triplicate (respectively, at 2 × 10^5^ or 1 × 10^6^ cells per 10-cm-diameter dish), infected with H-1PV (1 PFU/cell), and cultured for up to 120 h. Control (mock-infected) cells were harvested at the corresponding time points. Cells were washed twice with PBS and then fixed with 0.7 mL ice-cold 100% ethanol and 0.2 mL PBS. Fixed cells were stored at −20 °C. For analysis, the cells were pelleted for 10 min at 400× *g* and 4 °C and washed twice with PBS. Cell pellets were resuspended in PBS containing 100 mg/mL RNase H and 5 μg/mL propidium iodide (Sigma-Aldrich Inc., St. Louis, MO, USA). The stained cells were filtered through a 41-μm nylon mesh, incubated on ice for 1 h in the dark, and their DNA content was measured on a FACSort flow cytometer (Becton, Dickinson and Company, Franklin Lakes, NJ, USA). A minimum of 10,000 events were recorded and analyzed with the Cell-Quest^TM^ software (from Becton, Dickinson, NJ, USA). Differences in cell cycle distribution were tested for statistical significance by a two-sided Student’s *t*-test.

## 3. Results

### 3.1. Protoparvovirus H-1PV Enters and Transduces Non-Transformed and Transformed Mesenchymal Cells

**Figure 1:**
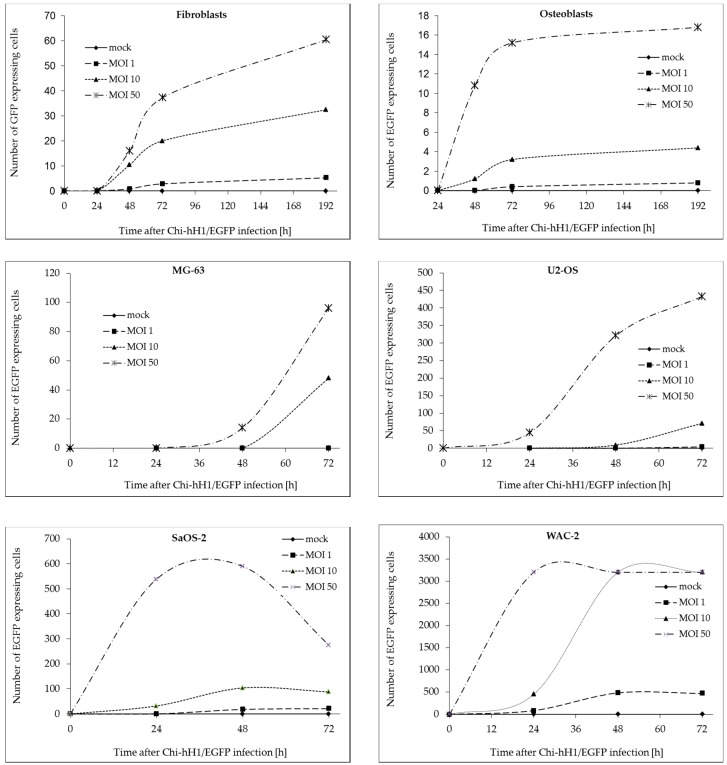
Susceptibility of bone tumor cells to H-1PV infection. Primary human osteoblasts and fibroblasts, the human osteosarcoma cell lines U-2 OS, SaOS-2, and MG-63, and the human neuroblastoma cell line WAC-2 were infected with Chi-hH1/EGFP at a multiplicity of infection (MOI) of 0, 1, 10, or 50 RU/cell. Green florescent cells were counted at different times post-infection. Counting was done manually in a pool of 4000 (for cell lines) or 10,000 cells (for primary osteoblasts or fibroblasts) per well.

We first determined whether H-1PV could enter mesenchymal cells and express its viral genes. For this, cultures of a panel of cells were inoculated with a recombinant replication-deficient H-1PV-based vector expressing EGFP (Chi-hH1/EGFP). To investigate whether parvovirus infection of mesenchymal cells depended on the transformation status of the cells or on an osteogenic-lineage-specific differentiation pattern, viral infectivity was compared in primary human osteoblasts, non-transformed neonatal foreskin fibroblasts, and three osteosarcoma cell lines. The neuroblastoma cell line WAC-2, a SH-EP subclone stably transfected with the *MYCN* gene, was used as a positive control [39]. Cells were infected with Chi-hH1/EGFP at an MOI of 0, 1, 10, or 50 replication units (RU) per cell, and EGFP-positive cells were counted manually under a fluorescence microscope at 24-h intervals for 3 days after infection (Figure 1). In the case of primary osteoblasts and fibroblasts, the observation period was extended to 192 h post-infection (Figure 1), as these non-transformed mesenchymal cells displayed no relevant cytopathic effect over this timespan. In each cell culture the intensity of the fluorescence increased over time in the infected cells. The higher the efficacy of viral entry and transduction was, the earlier this maximum level of EGFP expression has been reached.

In WAC-2 viral gene and transgene expression reached its maximum within 24 h after infection, whereas in SaOS-2 48 h and in the other osteosarcoma cell lines 72 h are necessary to reach maximum EGFP expression levels. 

**Figure 2:**
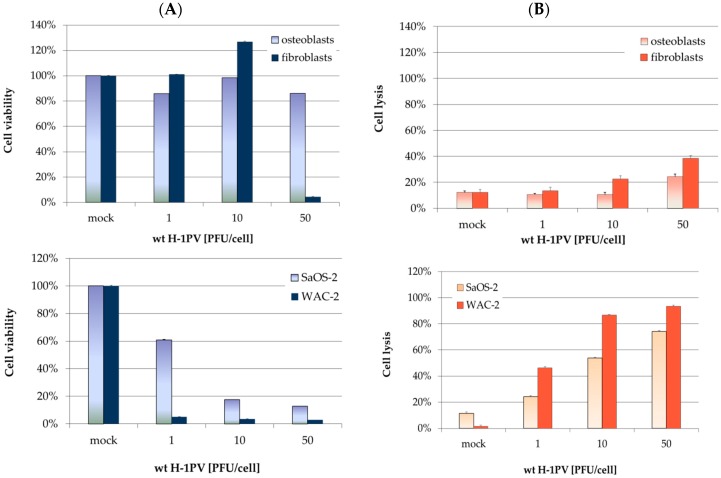
Innocuousness of wild-type H-1PV towards non-transformed mesenchymal cells. (**A**) 3-(4,5-dimethylthiazol-2-yl)-2,5-diphenyltetrazolium bromide (MTT) test for cell viability quantification, performed 7 days after H-1PV infection. Metabolic activity values were normalized to mock-treated control cells. Bars represent means of eight independent experiments, and the corresponding error bars represent standard errors of the mean (SEM); (**B**) lactate dehydrogenase (LDH)-release assays for quantifying H-1PV-induced cell lysis. LDH release was assayed three days after H-1PV infection. LDH activity in supernatants was normalized with respect to complete lysis with Triton. SaOS-2 and WAC-2 (lower panel) were used as positive controls.

As expected, the non-transformed cells showed low permissiveness towards H-1PV infection [23]. At the highest MOI tested (50 RU/cell), fluorescence was observed in only 4‰ of primary osteoblasts and 15‰ of human neonatal fibroblasts. EGFP expression persisted throughout the 8-day post-infection observation period (Figure 1, upper panel). It is worth noting that upon infection with H-1PV at MOI ≥ 50 PFU/cell, proliferation of human foreskin fibroblasts was inhibited (Figure S1), and their lysis induced (Figure 2). The effect of the virus on primary human osteoblast integrity and viability was likewise marginal (Figure S1). In other words, virus-induced cytotoxicity towards non-transformed mesenchymal cells was limited. The different osteosarcoma cell lines also showed differences in susceptibility to H-1PV infection. MG-63 displayed the lowest susceptibility, with only 2.5% EGFP-expressing cells 72 h after infection at MOI = 50 RU/cell. Under the same conditions, the percentage of fluorescent cells was 10% for the U2-OS cell line and 15% for the SaOS-2 cell line. In the WAC-2 cell line used as positive control, the proportion of fluorescent cells after 72 h was 80% after infection at MOI = 10 RU/cell.

The effects of H-1PV infection in non-transformed mesenchymal cells were further characterized and quantified as regards cell viability (MTT tests) and virus-induced cell lysis (LDH-release assays) shown in Figure 2. After infection with high titers of virus, neonatal fibroblasts appeared more susceptible to virus-induced cytotoxicity than osteoblasts (Figure 2, right upper panel). When the former cells were infected with H-1PV at an MOI of 50 PFU/cell, a highly significant reduction in mitochondrial metabolism was observed (Figure 2, left upper panel). This reduction in cell viability correlated with lysis of a significant number of neonatal fibroblasts. Osteoblasts, on the other hand, showed no substantial reduction of metabolic activity for up to 7 days post-infection, even after infection at an MOI of 50 PFU/cell. A 10% decrease in metabolic activity was observed, accompanied by a corresponding increase in cell lysis. In contrast, SaOS-2 and WAC-2 cells showed a clear, dose-dependent cytopathic effect within 72 h after infection (Figure 2, lower panel).

### 3.2. H-1PV Infection of Non-Transformed Mesenchymal Cells Induces Antiproliferative Effects and Toxicity Only at High Virus Doses

As mentioned above, no cytopathic effects were observed in primary osteoblasts or neonatal foreskin fibroblasts after infection with Chi-hH1/EGFP at an MOI up to 50 RU/cell. To determine the therapeutic window of wild-type H-1PV application to mesenchymal cells, cells were infected with this virus at increasing MOI. Although no cytopathic effect was observed after infection of osteoblasts or fibroblasts with low doses of H-1PV, the number of cells was found to decrease within 7 days after infection at MOI = 50 PFU/cell or higher. These cytomorphological findings indicate that at high MOI, wild-type H-1PV infection does have a significant cytotoxic effect on these non-transformed cells (Figure S1). In a clinical setting, however, such high MOIs are unlikely to be reached in replication-deficient, non-transformed tissues, if not caused by local, iatrogenic infections.

### 3.3. H-1PV Viral Protein Expression Induces Cell Cycle Arrest in G2/M in Osteosarcoma Cells

**Figure 3:**
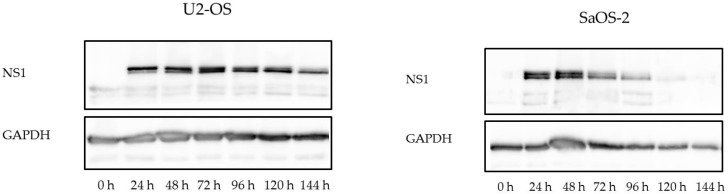
Western blot analysis of NS1 in H-1PV-infected osteosarcoma cells. Osteosarcoma cells were either mock-treated or infected with wild-type H-1PV (MOI: 1 plaque-forming units (PFU) per cell). Cell lysates from cultures collected at the indicated times were processed for western blotting with NS1-specific primary antiserum and horseradish-peroxidase-conjugated secondary antibodies. Blots were revealed by chemiluminescence. In H-1PV-infected cell lysates, two NS1-specific bands of approximately 60 kDa were detected. GAPDH was used as loading control. Results for SaOS-2 and U2-OS are shown as examples.

**Figure 4:**
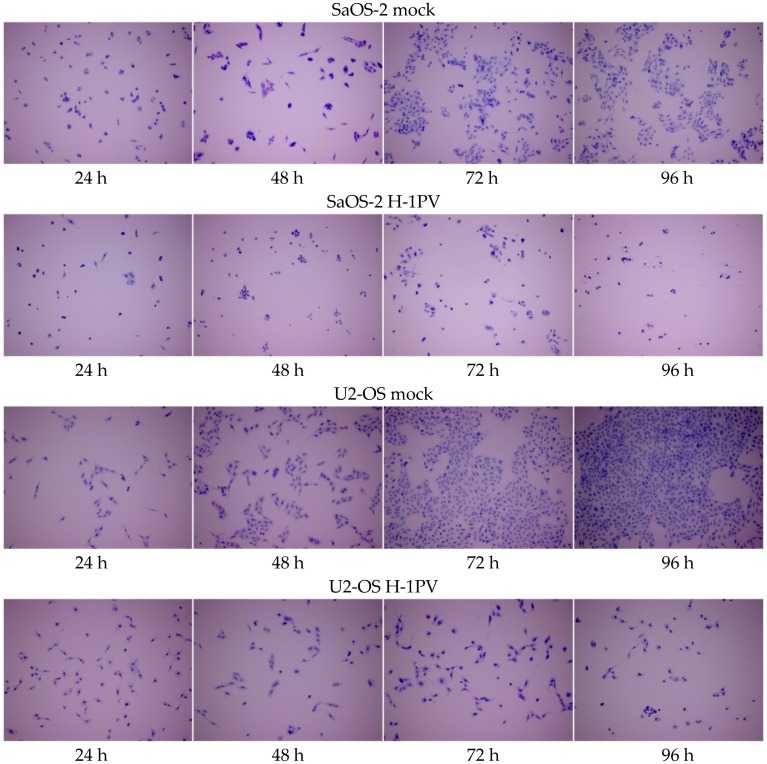
H-1PV infection induces antiproliferative and cytotoxic effects in osteosarcoma cell lines. Microscope images of osteosarcoma cells stained with crystal violet at 24-h intervals for up to 96 h after infection with 1 PFU wild-type H-1PV per cell. Upper panels: mock-infected cells. Magnification: 100×.

Expression of the nonstructural protein NS1 encoded by wild-type H-1PV was analyzed in a time course experiment by western blotting (Figure 3). In all osteosarcoma cell lines, NS1 accumulation became detectable between 12 and 24 h after H-1PV infection, reached a maximum after 48 h, and started to decrease 120 h post-infection in U2-OS cells (Figure 3, left part) or 72 h post-infection in SaOS-2 cells (Figure 3, right part). In all cases, the decrease was slow until about 80% of the cells in the culture had died. For each cell line, the time point at which a significant decrease in NS1 expression was detected correlated with the observation of distinct cytomorphological changes indicating a cytopathic effect of H-1PV in the cell line considered (Figure 4 and Figure S2).

**Figure 5:**
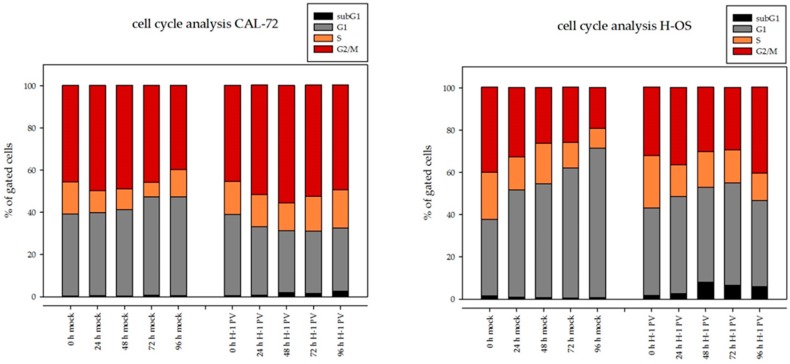

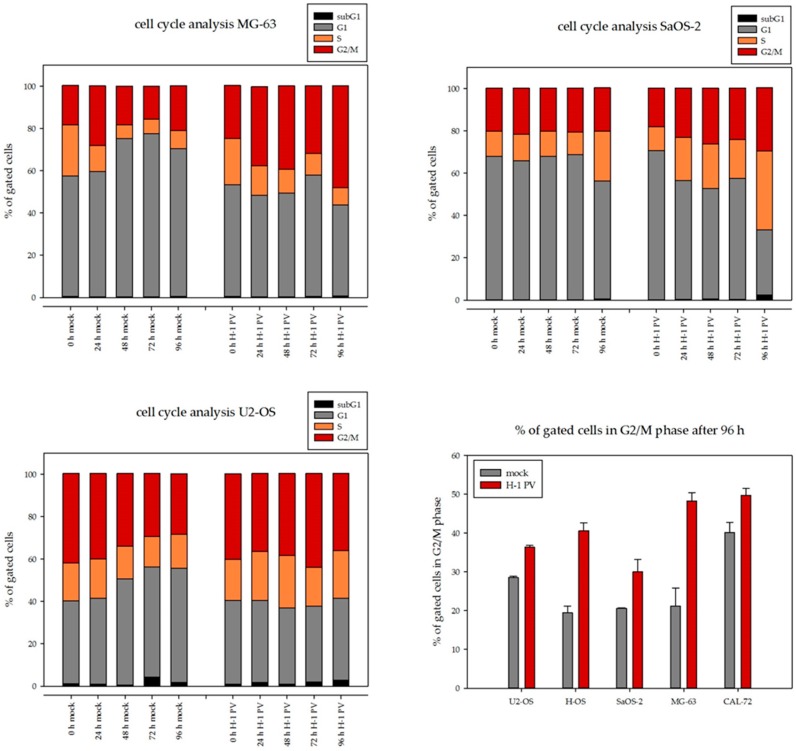
H-1PV infection induces G2/M arrest in osteosarcoma cells. FACSort flow cytometer analysis after propidium iodide staining. Acquisition of at least 10,000 events for each flow cytometric analysis allowed calculating the percentage of cells in G1, S, and G2/M at each time point (left panel). An excerpt focusing on mock- and H-1PV-infected cells arrested in G2/M at the time of infection and 96 h thereafter is provided in the right lower panel. Increase of cells in G2/M in H-1PV infected cells in comparison to mock-treated cells is highly significant (*p* > 0.001, two-sided Student’s *t*-test) for each cell line.

Flow cytometric analyses revealed, 96 h after H-1PV infection, a significant (10–25%) decrease in cells in G1 in all osteosarcoma cell lines tested. Simultaneously, a significantly higher proportion of cells in G2/M (10–30%) were observed in each cell line, as compared to mock-infected cells. H-1PV infection did not increase the percentage of apoptotic cells (subG1 fraction) in the osteosarcoma cell lines analyzed. The capacity of the H-1PV NS1 protein to disrupt the cell cycle has been described in an NS1-inducible stable HEK293-derived cell line [40]. We next investigated the effects of prolonged NS1 expression on the cell cycle distribution of osteosarcoma cells. After infection, cells were collected at 24-h intervals over a 96-h period, as described for the western blot analysis. Cell cycle analyses were performed at each time point. Percentages of cells in the different phases of the cell cycle are represented in Figure 5. 

### 3.4. Osteosarcoma Cells Undergo Lytic H-1PV Infection

Virus-induced cytopathic effects (CPE) on osteosarcoma cells were visualized by crystal violet staining of the cells at different time points after H-1PV infection. They were observed in all established osteosarcoma cell lines, and the number of cells affected increased over time. In CAL-72, H-OS, SaOS-2, and U2-OS cells, H-1PV-induced cytomorphological changes appeared 48 h after infection. In all osteosarcoma cell lines, H-1PV infection reduced cell proliferation and induced significant cell death, as shown for SaOS-2 and U2-OS (Figure 4) and as documented in Figure S2 for the CAL72, H-OS, and MG-63 cell lines. In MG-63 cells, the onset of virus-induced CPE became obvious 72 h after infection.

### 3.5. In Osteosarcoma Cells, H-1PV Infection Does Not Lead to Efficient Production of Infectious Viral Progeny

The capacity of the autonomous protoparvovirus H-1PV to replicate in malignant cells has been widely described [23,28]. Yet human malignant cells of different origins are known to vary in their capacity to produce fully infectious viral progeny, and one might expect the antitumor efficacy of H-1PV to increase with the ability of the infected cells to produce and spread progeny virions. Therefore, virus replication was determined in our panel of osteosarcoma cell lines. In time-course experiments performed as described in the Materials and Methods section, the increase in full particles and the release of infectious viral particles into the supernatant were quantified in each of the five established osteosarcoma cell lines.

**Figure 6:**
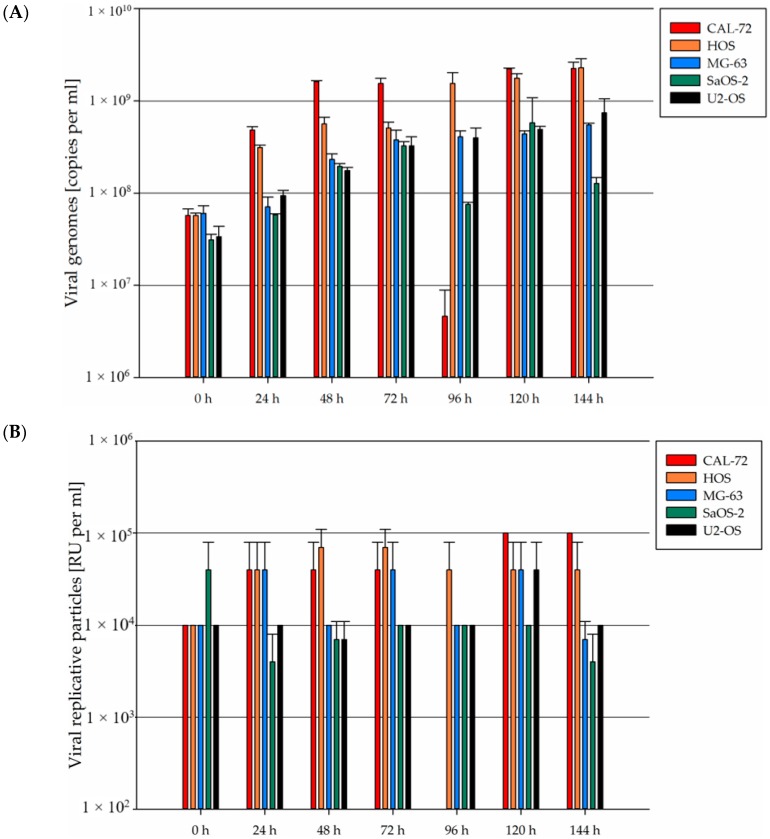
The efficiency of wtH-1PV replication in osteosarcoma cells is limited. Cells were infected with one infectious particle per cell (PFU/cell) and cell supernatants were collected at the indicated times. (**A**) The number of viral genome copies released was determined into the cell culture medium in triplicate by quantitative polymerase chain reaction (qPCR) (copies/mL), and means are shown. Error bars represent SEM (upper panel); (**B**) titers of infectious particles (RU/mL) were determined by infecting NBK324K cells with serial dilutions of the cell culture supernatant and by dot blot detection of viral DNA 72 h after infection. Error bars represent SEM (lower panel).

Compared to the input virus remaining in the cell culture medium (0 h), the number of viral DNA copies increased 5- to 10-fold in all five osteosarcoma cell lines, as determined by quantitative PCR (Figure 6, upper panel). Accordingly, the titer of full infectious viral particles did not increase by more than one log-step over the 144 h following infection in any of the five osteosarcoma cell lines tested (Figure 6, lower panel).

### 3.6. Wild-Type H-1PV Is More Cytotoxic Towards Osteosarcoma Cells Than H-1PV-Derived Fitness Mutants

Given the limited replicative capacity of wild-type H-1PV in osteosarcoma cells, we tested whether higher cytotoxicity might be achieved after infecting osteosarcoma cells with one of two derived virus mutants: Del H-1PV, a virus mutant previously described to propagate more efficiently in human pancreatic cancer cells [32], and H1-DM, a virus fitness mutant carrying two point mutations in the non-structural (NS) coding gene. The cytotoxic effects on osteosarcoma cells of the two mutant viruses and of wild-type H-1PV were compared. To assess cell viability, MTT tests and quantification of metabolic activity in surviving cells were performed. To quantify virus-induced osteosarcoma cell lysis, LDH-release assays were carried out. LD50 titers of input virus were determined as described in the Materials and Methods section.

**Figure 7:**
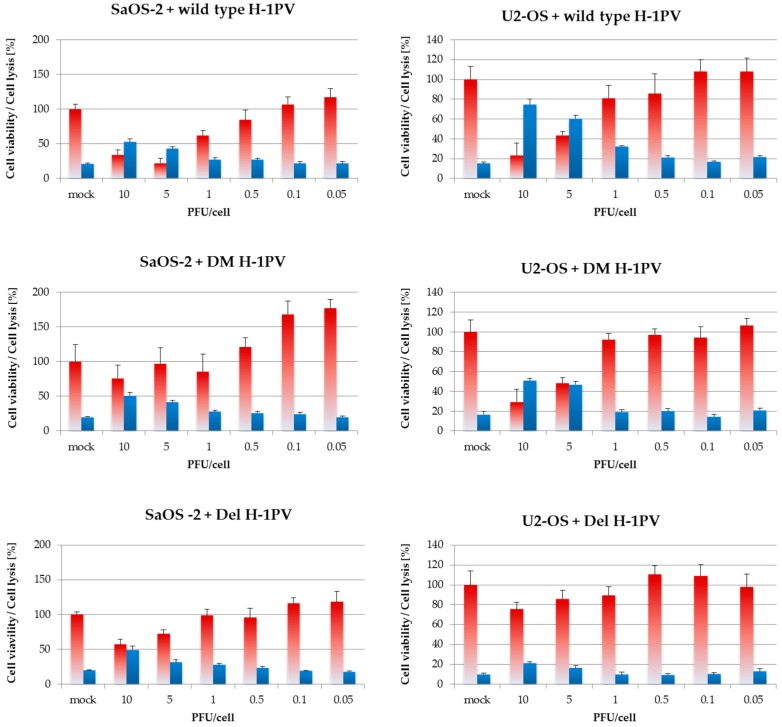
Six days after infection, wild-type H-1PV shows stronger toxicity towards osteosarcoma cells than mutant viruses. Cells were infected with increasing titers of wild-type H-1PV, Del H-1PV, or double-mutant H1-DM. Six days after infection, cytotoxicity testing was performed in 96-well plates in simultaneous MTT tests and LDH-release assays. Red and blue bars represent means of eight independent experiments and error bars represent standard errors of the mean (SEM). Cell viability is indicated in blue and cell lysis in red.

As exemplified in Figure 7 and Figure S3 and as quantitated in Table 1, Del H-1PV showed the lowest cytotoxicity in all osteosarcoma cell lines and did not induce any significant cytotoxic effects in two cell lines, MG-63 and U2-OS, within the range of doses tested. In CAL-72, for example, the LD50 for Del H-1PV was 10 times as high as for DM H-1PV and 20 times as high as for wild-type H-1PV. For DM H-1PV, the LD50 values determined ranged from 1 PFU per cell in CAL-72 to 25 PFU per cell in MG-63. In other words, the dose of input virus required to induce lytic infection of at least 50% of the osteosarcoma cells was the same (in U2-OS) to 5 times as high (in H-OS) with DM H-1PV as with wild-type H-1PV. In these experiments, wild-type H-1PV thus induced osteosarcoma cell death at a lower dose of input virus than did either of the two mutants tested.

LD50 of input virus as determined on day 6 after infection with wild-type H-1PV or a mutant virus. Cytotoxic effects induced by infection with wild-type H-1PV, the DM H-1PV double mutant, or the Del H-1PV mutant (having a deletion in NS) were quantified by assaying cell viability and cell lysis as described in the Materials and Methods section. Briefly, 6 days after infection with a viral strain at increasing MOI, MTT tests and LDH-release assays were performed in parallel. LD50 of input virus are given in plaque-forming units per cell. If the LD50 of the tested virus strain was not reached under the conditions analyzed, “n. r.” is indicated.

## 4. Discussion

### 4.1. Safety Profile of Wild-Type H-1PV in Human Mesenchymal Cells

The protoparvoviruses, first described as H viruses, induce a characteristic pathogenesis in some rodent species. Transplacental infection or direct inoculation of H viruses into newborn hamsters affects replicating cells of the developing bone. Infection of these cells results in dwarfism and malformation of teeth and bones in surviving hamsters. This characteristic phenotype has been described as osteolytic syndrome [41]. Inoculation of H-1PV within the first 48 h postpartum most consistently induces the complete phenotype. Animals infected at a later time show fewer signs the older they are at the time of infection. H-1 dr, the first variant H-1 virus described to have an increased capacity to replicate in human cells, also shows this specific pathogenicity in newborn hamsters [42]. Signs of an osteolytic syndrome have never been reported after infection of adult hamsters by rodent parvoviruses [26,41]. In rats, the natural host of H-1PV, virus-induced pathogenicity shows the same age-dependency: in fetuses and neonates, H-1PV infection can be pathogenic or even lethal, whereas in adult animals it remains clinically unapparent [26]. These observations have been taken as evidence that parvovirus pathogenicity is restricted to proliferating and differentiating mesenchymal tissues. 

Therefore, prior to a clinical application in child or adolescent sarcoma patients, it was important to evaluate the cytotoxicity of H-1PV very carefully in primary mesenchymal cells of pediatric origin. Our results show that H-1PV can enter non-transformed mesenchymal human cells, such as osteoblasts and fibroblasts. Yet in our study, the only non-transformed cells to be transduced were neonatal human fibroblasts, and only after infection at high MOI they did show a clear cytopathic effect. Such high viral titers are unlikely under conditions other than local infections of neighboring replication-competent cells or local virus applications in the course of treatment. The virus dose range at which H-1PV is non-toxic towards non-transformed mesenchymal cells is comparable to the dose range previously identified as innocuous towards non-transformed infant neuroepithelial cells [39].

### 4.2. Osteosarcoma Cells Are Semi-Permissive to Wild Type H-1PV Infection

Oncolytic effects induced by H-1PV have been observed in a variety of rodent and human cancer cells [26]. A previous publication has described a parvovirus-H1-derived vector expressing Apoptin to have a greater capacity to induce apoptosis than wild-type H-1PV [43]. In proof-of-concept experiments in which wild-type H-1PV was applied to a set of four cell lines, including two osteosarcoma cell lines, first indications of the capacity of wild-type H-1PV to induce cytopathic effects and subsequent cell death were obtained [43].

As a first step in assessing the therapeutic potential of H-1PV for pediatric and adolescent osteosarcoma, the aim of the present study was to assess the capacity of H-1PV to replicate in osteosarcoma cell lines and to perform a systematic quantification in vitro of H-1PV-induced effects on the cell cycle and cell viability. In the five pediatric osteosarcoma cell lines analyzed, accumulation of the major parvoviral cytotoxic protein NS1 started during the first 12–24 h after infection with wild-type H-1PV, and the amount accumulated remained unaltered for at least 120 h. NS1 has been shown to be sufficient to induce G2-arrest in HEK-293 and HeLa cells. In these cells, G2 arrest leads to activation of caspases 9 and 3 which results in apoptotic cell death [40].

In all osteosarcoma cell lines, a significant increase in the proportion of H-1PV-infected cells arrested in G2/M was observed over time. The observed virus-induced disturbances of the cell cycle were accompanied by distinct cytomorphological changes in all osteosarcoma cell lines. Eventually, osteosarcoma cell proliferation was inhibited and cell death occurred. In contrast to findings on pediatric neuroectodermal tumor cells [39,44], apoptotic DNA fragmentation does not appear to play a key role in this process in osteosarcoma cells, but the molecular mechanisms by which NS1 expression induces cell death in osteosarcoma remain to be further characterized.

Efficient spreading of an oncolytic virus throughout a tumor mass requires the capacity to induce secondary rounds of infection. Essential prerequisites to this process are efficient virus replication and viral egress. Although wild-type H-1PV has been characterized as a self-replicating oncolytic virus, its replication efficiency varies widely among different tumor entities [26]. We therefore quantified the virus production capacity of the cell lines studied. Over a seven-day period post-infection, only two cell lines showed an increase in infectious particle titer, and this increase was limited. This poor viral replication efficiency may limit the therapeutic efficacy of wild-type H-1PV in osteosarcoma patients.

### 4.3. Fitness Mutants Do Not Show Increased Cytotoxicity Towards Osteosarcoma Cells In Vitro

Two mutant protoparvovirus H-1 strains, Del H-1PV [32] and DM H-1 [45], have been described as more efficient than wild-type H-1PV in progeny virus release and propagation in human cancer cell cultures. We have applied these strains to a panel of osteosarcoma cell lines characterized as semi-permissive to wild-type H-1PV infection, and have estimated their capacity to induce sustained cytotoxic effects. Both primary and secondary cytotoxic effects are observed after six days of in vitro exposure to low titers of input virus, depending on the efficiency of virus replication and propagation in the transformed host cell population through successive rounds of infection. The first fitness mutant derived from standard H-1PV, H-1 dr, was described in 1995 [42]. H-1 dr is a naturally occurring variant with a higher efficiency of spreading in permissive human cells. This variant is characterized by specific sequence alterations, including an in-frame deletion of codons 39 to 41 and a tandem duplication of 58 nt close to the right-hand origin of replication. The deletion in the region encoding NS1 and NS2 results, upon translation, in truncated viral proteins [42]. Del H-1 was derived from the existing molecular standard H-1PV clone by deleting nucleotides 2022 to 2135 [32], so as to mimic the 114-nt deletion occurring naturally in H-1dr. The truncated NS1 and NS2 proteins are hypothesized to play a key role in the greater capacity of Del H-1 progeny virions to be exported from the nucleus and to egress from the host cell, and hence in the increased antineoplastic efficacy of the mutant virus in two pancreatic cancer xenograft models [42].

In our comparative analysis of viral cytotoxicity towards the panel of osteosarcoma cell lines, focusing on the wild-type virus and the above-mentioned variants, Del H-1 required the highest dose of input virus to cause at least 50% of the cell population to die. In two osteosarcoma cell lines, Del H-1PV was unable to induce any significant cytotoxic effect, even at the highest MOI applied. With the fitness mutant H-1 DM, the LD 50 of input virus was reached in all five osteosarcoma cell lines. It ranged from 1 PFU per cell in the most susceptible cell line (CAL-72) to 50 PFU per cell in the least susceptible cell line (MG-63). The highest efficacy against semi-permissive pediatric osteosarcoma cells was observed with the standard wild-type H-1PV: in all five osteosarcoma cell lines tested, the LD50 titer of input wild-type H-1PV ranged from 0.5 to 10 PFU/cell. These unexpected in vitro data led us to speculate that the previously described higher replication and spreading efficiency of the mutant viruses Del H-1PV and H-1 DM might be restricted to virus-replication-competent host cells. Our experiments performed to define the therapeutic window for wild-type H-1PV showed it to be innocuous to non-transformed mesenchymal cells at MOIs below 50 PFU/cell. Accordingly, the cytotoxic effects of standard H-1PV measured in osteosarcoma cell culture models correspond to a therapeutic index of 5 to 100.

The present study is part of a strategy aiming to define pediatric indications for first applications of H-1PV in clinical trials testing the therapeutic efficacy of H-1-based parvovirotherapy. We show that osteosarcoma is a potential parvovirus-responsive pediatric tumor entity, as all the human sarcoma cells tested proved sensitive to the cytopathic effects of wild-type H-1PV. To take full therapeutic advantage of the application of a self-replicative oncolytic virus, it may be desirable to select target tumors that show some degree of competence for virus multiplication and propagation. Our data show that human osteosarcoma cells are heterogeneous in this regard and fail to achieve high yields of progeny H-1PV production. In an attempt to overcome this limitation, we have tested two H-1PV mutants reported to replicate more efficiently than H-1PV in other human cancer cell models, but these mutants failed to surpass the wild-type parental virus in osteosarcoma cells. This illustrates the host-cell-type specificity of their enhanced infectivity.

## 5. Conclusions

The present in vitro study represents a systematic preclinical analysis, in pediatric and adolescent osteosarcoma cell culture models, of the oncoselectivity and oncolytic efficacy of the wild-type protoparvovirus H-1PV and of its mutant derivatives Del H-1PV and H-1DM. We show that, in contrast to observed effects on other malignant diseases, the wild-type protoparvovirus H-1PV eliminates osteosarcoma cells more effectively than the two mutant viruses. Future steps in the preclinical validation of H-1PV for osteosarcoma treatment will include, besides adapting the virus for enhanced production, combining it with other cancer therapeutics in the framework of multimodal treatment strategies. Systematic preclinical testing should pave the way towards well-designed clinical trials evaluating oncolytic parvovirotherapy in child, adolescent, and young adult osteosarcoma patients.

## Figures and Tables

**Table 1:** LD50 titers of wild-type H-1PV and mutant virus strains in pediatric osteosarcoma cell lines.

Cell Culture	Del H-1PV	DM H-1PV	wt H-1PV
CAL-72	10	1	0.5
H-OS	5	5	1
MG-63	n. r.	25	10
SaOS-2	>10	10	5
U2-OS	n. r.	5	5

## Supplementary Materials

Supplementary materials can be found at www.mdpi.com/1999-4915/9/10/301/s1.
