# Supplementary material for: Preclinical Testing of an Oncolytic Parvovirus: Standard Protoparvovirus H-1PV Efficiently Induces Osteosarcoma Cell Lysis In Vitro
Source: Viruses. 2017 Oct 17;9(10):301. doi: 10.3390/v9100301 (PMC5691652; doi:10.3390/v9100301)
Supplement: Supplementary file 1 [file viruses-09-00301-s001.pdf]

Supplementary Materials: Preclinical Testing of an Oncolytic Parvovirus: Standard Protoparvovirus H-1PV Efficiently Induces Osteosarcoma Cell Lysis In Vitro

Carsten Geiss, Zoltán Kis, Barbara Leuchs, Monika Frank-Stöhr, Jörg R. Schlehofer, Jean Rommelaere, Christiane Dinsart and Jeannine Lacroix

Table S1. Human osteosarcoma cell lines used in this study.

Cell Culture	Age	Gender	Year	Morphology	Reference
CAL72	10	male	1989	fibroblastoid	[1]
H-OS	13	female	1971	mixed*	[2]
MG-63	14	male	1977	fibroblastoid	[3]
SaOS-2	11	female	1973	epithelial	[4]
U2-OS	15	female	1964	epithelial	[5]

* mixed, fibroblast and epithelial like morphology

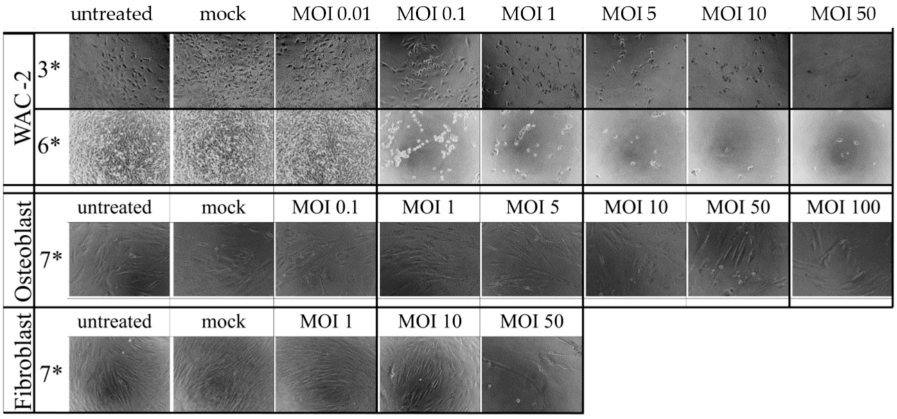

Figure S1. No visible cytopathic effects of wild type H-1PV in non-transformed mesenchymal cells. Phase contrast microscopy images for evaluating the effect of H-1PV dose on cell morphology. Images were recorded prior cell viability and cytotoxicity testing at day 7 in case of primary fibroblast and osteoblast cells and in WAC-2 cells at days 3 and 6 after H-1PV infection (400× magnification).

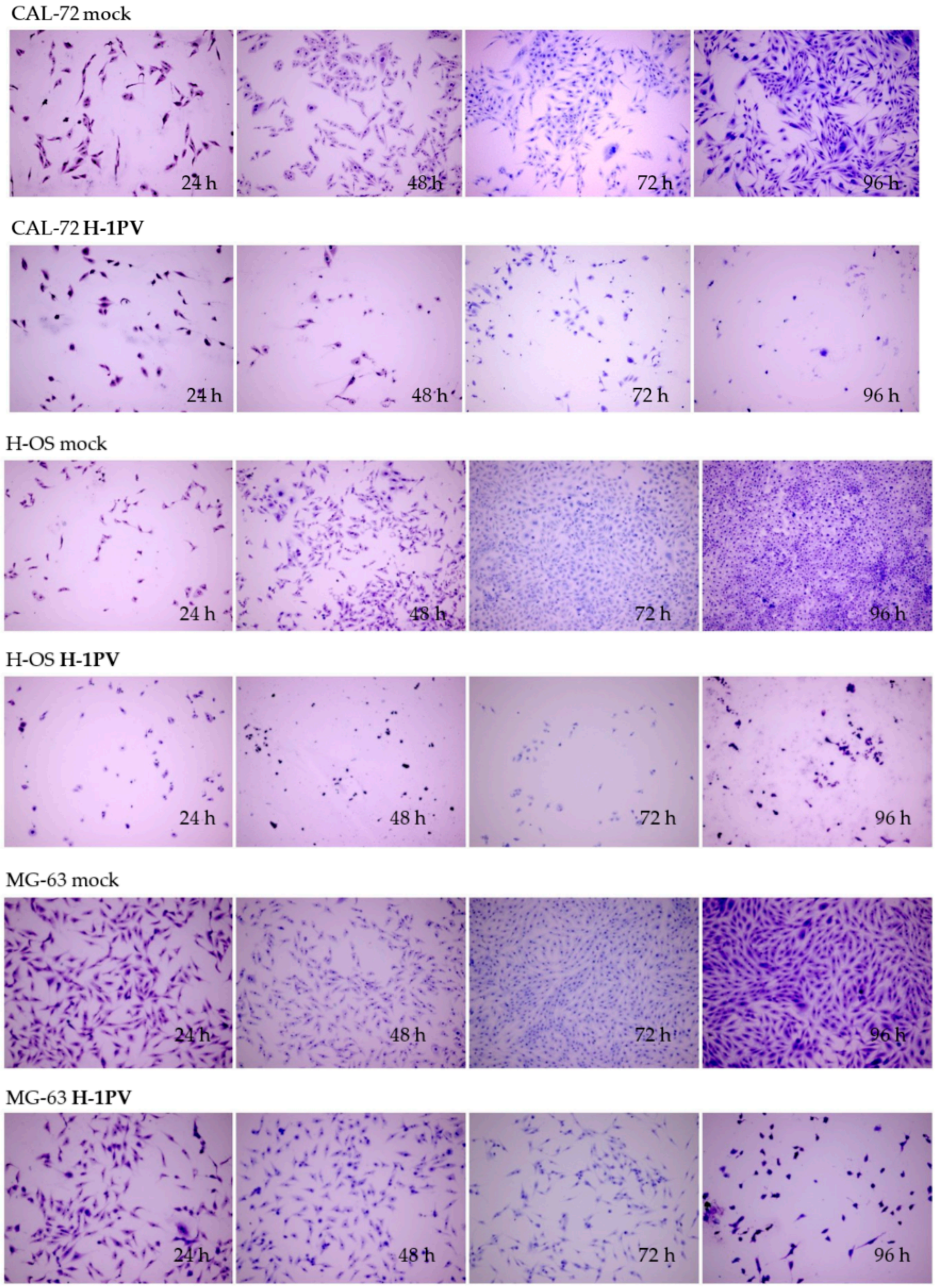

Figure S2. H-1PV infection induces antiproliferative and cytotoxic effects in osteosarcoma cell lines (continued from Figure 5).

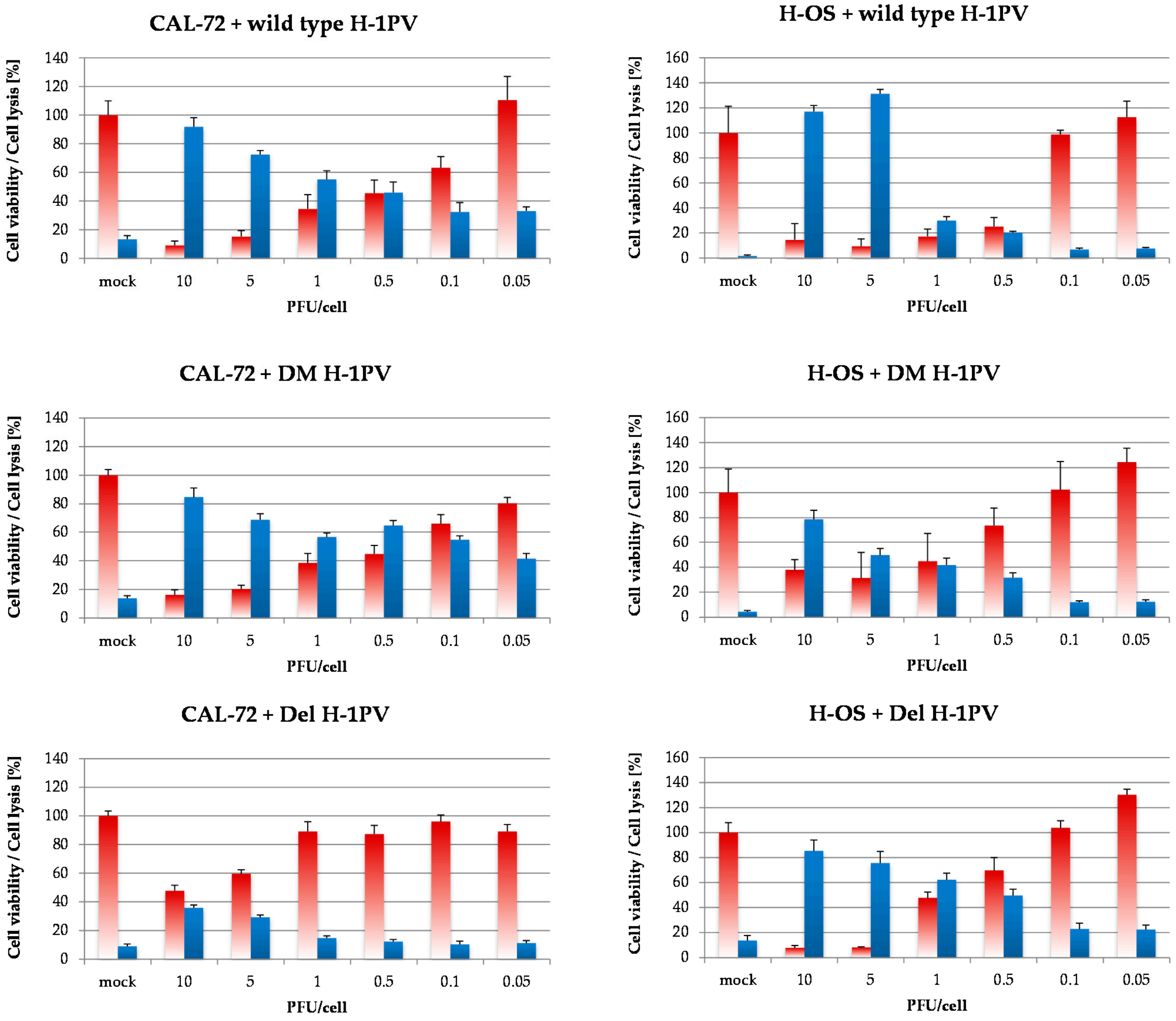
CAL-72 + wild type H-1PV
Cell viability / Cell lysis [%]
0 20 40 60 80 100 120 140
mock 10 5 1 0.5 0.1 0.05
PFU/cell
H-OS + wild type H-1PV
Cell viability / Cell lysis [%]
0 20 40 60 80 100 120 140 160
mock 10 5 1 0.5 0.1 0.05
PFU/cell
CAL-72 + DM H-1PV
Cell viability / Cell lysis [%]
0 20 40 60 80 100 120 140
mock 10 5 1 0.5 0.1 0.05
PFU/cell
H-OS + DM H-1PV
Cell viability / Cell lysis [%]
0 20 40 60 80 100 120 140 160
mock 10 5 1 0.5 0.1 0.05
PFU/cell
CAL-72 + Del H-1PV
Cell viability / Cell lysis [%]
0 20 40 60 80 100 120 140
mock 10 5 1 0.5 0.1 0.05
PFU/cell
H-OS + Del H-1PV
Cell viability / Cell lysis [%]
0 20 40 60 80 100 120 140 160
mock 10 5 1 0.5 0.1 0.05
PFU/cell

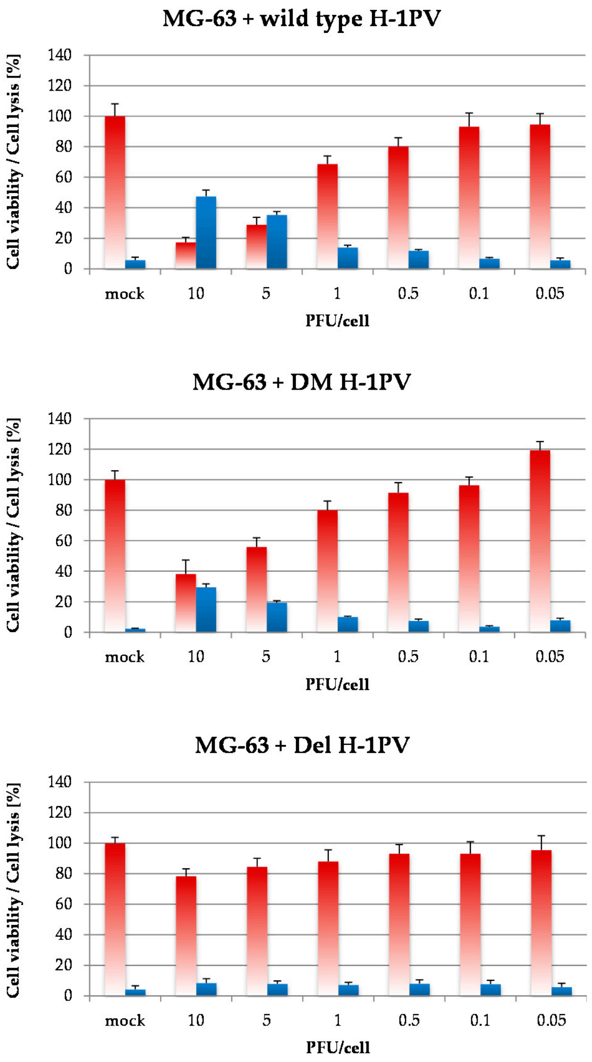

Figure S3. Comparative cytotoxicity testing on mutant H-1PV strains Del H-1PV and DM H-1PV, and standard wild type H-1PV (continued from Figure 7).

References

1. Rochet, N.; Dubousset, J.; Mazeau, C.; Zanghellini, E.; Farges, M.F.; de Novion, H.S.; Chompret, A.; Delpech, B.; Cattan, N.; Frenay, M.; et al. Establishment, characterisation and partial cytokine expression profile of a new human osteosarcoma cell line (CAL 72). *Int. J. Cancer* **1999,** 82, *2*, 282–285.
2. McAllister, R.M.; Gardner, M.B.; Greene, A.E.; Bradt, C.; Nichols, W.W.; Landing, B.H. Cultivation in vitro of cells derived from a human osteosarcoma. *Cancer* **1971,** 27, *2*, 397–402.
3. Billiau, A.; Edy, V.G.; Heremans, H.; Van Damme, J.; Desmyter, J.; Georgiades, J.A.; De Somer, P. Human interferon: Mass production in a newly established cell line, MG-63. *Antimicrob. Agents Chemother.* **1977**, *12*, 11–15.

4. Fogh, J.; Wright, W.C.; Loveless, J.D. Absence of HeLa cell contamination in 169 cell lines derived from human tumors. *J. Natl. Cancer Inst.* **1977**, *58*, 209–214.
5. Ponten, J.; Saksela, E. Two established in vitro cell lines from human mesenchymal tumours. *Int. J. Cancer* **1967**, *2*, 434–447.

© 2017 by the authors. Licensee MDPI, Basel, Switzerland. This article is an open access article distributed under the terms and conditions of the Creative Commons Attribution (CC BY) license (http://creativecommons.org/licenses/by/4.0/).
